# Lumbo-pelvino-azetabuläres Alignment – Grundlagen und klinische Konsequenzen

**DOI:** 10.1007/s00132-022-04321-x

**Published:** 2022-10-12

**Authors:** Bernhard Heimkes, Nina Berger, Vincent Frimberger

**Affiliations:** Klinik für Kinderchirurgie, Sektion Kinder- und Neuroorthopädie, Kliniken Dritter Orden gGmbH, Menzinger Straße 44, 80638 München, Deutschland

**Keywords:** Azetabulum, Beckenanteversion, Beckenretroversion, Becken, Sagittale Balance, Acetabulum, Pelvic anteversion, Pelvic retroversion, Pelvis, Sagittal balance

## Abstract

Die Wirbelsäulenform wie auch die pelvine Ante‑/Retroversion eines Individuums werden durch seine angeborene, genetisch fixierte lumbosakrale Angulation bestimmt. Diese kann wenig aufwendig in der seitlichen Stehaufnahme des Patienten vermessen werden. In der Wirbelsäulenchirurgie existiert zum Thema eine große Anzahl von Originalarbeiten, in der Hüftchirurgie wurde die individuelle Beckenversion und ihre Konsequenzen für die azetabuläre Orientierung der Hüftpfanne bisher weniger beachtet.

Im vorliegenden Review werden bisherige Kenntnisse zum Zusammenhang zwischen lumbosakraler Angulation und pelviner Ante‑/Retroversion dargestellt. Es lassen sich hierbei vier anatomisch definierbare Beckentypen unterscheiden, wovon drei als fakultativ pathogen angesehen werden müssen. Klinische Konsequenzen ergeben sich für die Krankheitsbilder der Spondylolisthesis, des nichtspezifischen Kreuzschmerzes, der azetabulären Retroversion, der kongenitalen Hüftdysplasie sowie für die Pfannenpositionierung in der Hüftendoprothetik.

## Abstract (en)

The spinal shape and the pelvic ante-/retroversion of an individual are determined by its innate, genetically fixed lumbosacral angulation. This can be measured with little effort in the lateral standing radiograph of the patient. In spinal surgery, there are a lot of original papers on the topic. In hip surgery, however, the individual pelvic version and its consequences for the acetabular orientation have not received the same attention so far.

This review focuses on previous data on the relationship between lumbosacral angulation and pelvic ante-/retroversion. Four anatomically definable pelvic types can be distinguished; three of those have to be considered as facultatively pathogenetic. Clinical consequences arise for the clinical pictures of spondylolisthesis, non-specific lower back pain, acetabular retroversion and developmental dysplasia of the hip, as well as for acetabular cup positioning in total hip arthroplasty.

## Hinführung

Entscheidend für den Erwerb des aufrechten Ganges war, dass sich im Laufe der Evolution eine lumbosakrale Angulation ausbildete, die das Becken aus der ursprünglich fast waagrechten Position in die Vertikale brachte und der Lendenwirbelsäule eine lordotische Form gab. In Grundlagenarbeiten wurden hierzu die lumbopelvinen Messparameter Sacral Slope, Pelvic Tilt und Pelvic Incidence eingeführt, wobei letzterer sowohl die Wirbelsäulenform als auch die Beckenversion eines Individuums bestimmt. Dieser Zusammenhang zwischen der Form der Wirbelsäule sowie der Version des Beckens und der Pfanne muss bei Beckenosteotomien sowie in der Hüftarthroskopie und Hüftendoprothetik beachtet werden.

## Beckenposition und Schwerelot (Abb. 1a)

Stabiles Stehen ist nur dann möglich, wenn der in Höhe des fünften Lendenwirbelkörpers befindliche Massenschwerpunkt entlang des Schwerelots („gravity line“) über die Standfläche der Füße gerät. Stehaufnahmen in Kombination mit Fußdruckmessungen gesunder Probanden [26, 27, 29] zeigen, dass die Lage des Schwerelots überwiegend über die Position des Beckens reguliert wird [16, 31].

**Figure 1:**
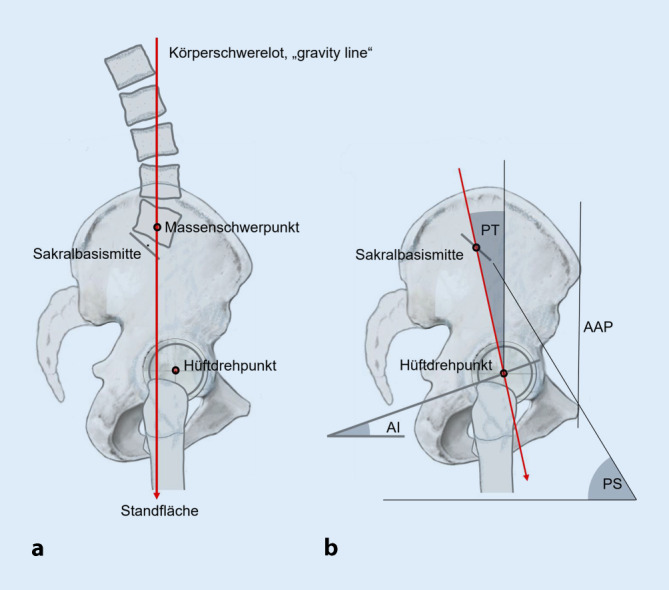

Das Vermögen, den Körper mithilfe der Propriozeption und geringen Muskelkräften möglichst ökonomisch in der Sagittalen zu stabilisieren, bezeichnet man als sagittale Balance.

## Sagittales Profil des Beckens, Messparameter (Abb. 1b, 2 und 3)

**Figure 2:**
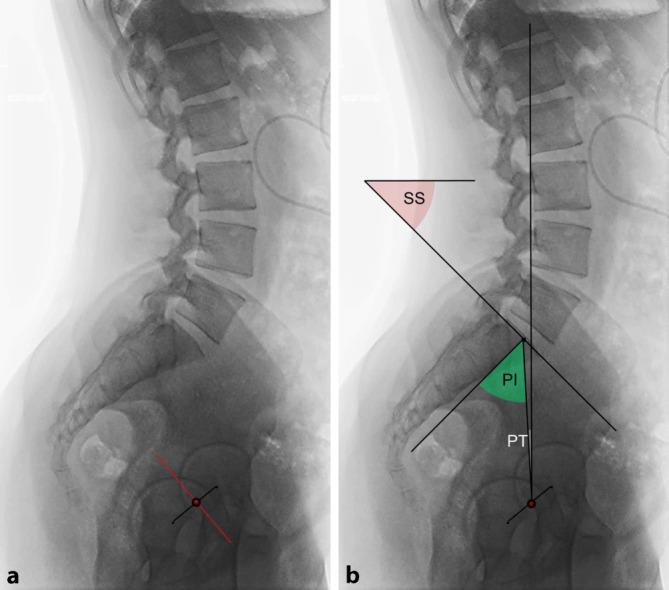

In der seitlichen Standaufnahme des Beckens, entweder konventionell radiologisch [4, 15, 25, 35] oder mit der EOS-Methode [2, 5, 8, 14, 16] gewonnen, kann das sagittale Profil des Beckens und damit der Becken-Tilt auf dreierlei Weise vermessen werden:

- Für endoprothetische Fragestellungen wird überwiegend die vordere Beckenebene (VBE, „anterior pelvic plane“ [AAP]) genutzt [19, 28, 36]. Sie ist durch eine Linie zwischen beiden Spinae iliacae anteriores superiores und den Schambeintuberkeln definiert. Die azetabuläre Anteversion (AI, Synonyme: Anteinklination, „acetabular cup anteversion“) ist bei fehlenden validen Messpunkten in der präoperativen Stehaufnahme nicht bestimmbar. Sobald die Hüftpfanne eingebracht ist, kann man jedoch mit diesem Wert die sagittale Position der Kunstpfanne und deren funktionelle Relativbewegungen im Liegen, Sitzen und Stehen vermessen.
- In radioanatomischen Arbeiten ist der Promontorium-Symphysen-Winkel (PS-Winkel, sagittaler Beckenneigungswinkel) aufgeführt [30, 34]. Er vermisst sich zwischen der Körperhorizontalen und einer Linie, die vom Promontorium zu den Schambeintuberkeln zieht. Als Normwert werden 60 ° angegeben, Untersuchungen zur Standardabweichung bestehen nicht.
- In der Wirbelsäulenchirurgie wird entsprechend der Abb. 2 und 3 das sagittale Profil des Beckens und des lumbopelvinen Überganges mit dem (nicht dargestellten) Lordosewinkel – bestimmt zwischen der Oberkante L5 und der Sakralbasis –, dem Sacral Slope (SS), der Pelvic Incidence (PI) und dem Pelvic Tilt (PT) beschrieben. Der Vorteil dieser Methode ist, dass in der Mitte der Sakralbasis alle Winkel zusammentreffen, die den Zusammenhang zwischen Wirbelsäulen- und Beckenprofil zeigen. Zudem bestehen mehrere konkordante Studien zu Durchschnittswerten und zur Korrelation zwischen Wirbelsäulen- und Beckenparametern [2, 4, 6, 8, 15, 16, 27, 35] Die zuletzt beschriebene Methode der Messung des Becken-Tilt wird zunehmend auch in der gelenkerhaltenden operativen Therapie der Hüfte [9, 10] wie auch in der Hüftendoprothetik verwandt [7, 13, 14].

**Figure 3:**
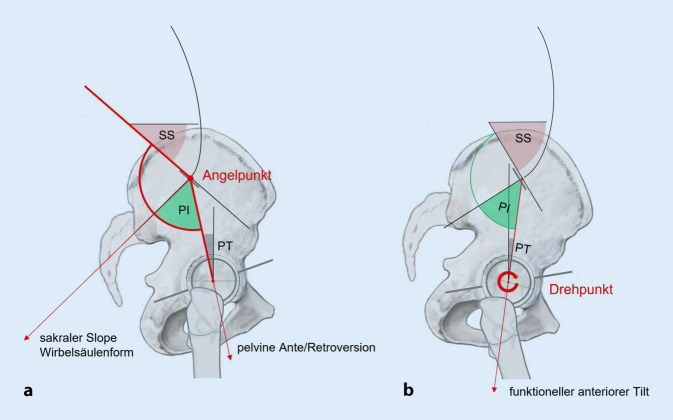

## Lumbopelvines Alignment (Abb. 3a, b)

Im sagittalen Profil der Wirbelsäule und des Beckens im Stand muss man unterscheiden, welche Werte im lumbopelvinen Alignment anatomisch vorgegeben, und welche Werte durch funktionelle Überlagerung entstanden sind. Es sind also Formfehler von Funktionsstörungen abzugrenzen.

Das lumbopelvine Alignment beschreibt die sich gegenseitig bedingende anatomische Form der Lendenwirbelsäule, des lumbosakralen Übergangs und des Beckens eines Individuums. Es ist evolutionär vorgegeben, genetisch determiniert und nach der Wachstumsphase lebenslang gleichbleibend, solange keine sekundär verformenden Krankheiten hinzukommen [4, 16, 25, 26, 31]. Wichtigster Parameter ist hierbei die Pelvic Incidence, deren unveränderlicher Wert vom jeweiligen Individuum abhängig ist. Diese gibt vor, wie stark die Sakralbasis innerhalb des Beckens anguliert ist, sie bleibt damit bei Vor- und Rückrotation im Hüftgelenk unverändert. Wie in Abb. 3a dargestellt, gibt ihr dorsaler Schenkel den Wert des Sacral Slopes vor, der wiederum die Wirbelsäulenform bestimmt [15, 25, 35]. Ihr ventraler Schenkel sagt aus, wieviel der anatomische Tilt des Beckens beträgt, also wie stark das Becken retro- oder antevertiert ausgebildet ist [2, 4, 8, 16, 35]. Wie in Abb. 3b dargestellt, ändert sich diese anatomisch vorgegebene Ausgangssituation des aufrecht stehenden Menschen, sobald zusätzlich zur formbestimmenden lumbosakralen Angulation eine (funktionelle) Vor- oder Rückrotation im Hüftgelenk vollzogen wird. Hierauf wird nochmals ausführlich im Folgekapitel „Funktionelle Änderungen der Beckenposition“ eingegangen.

## Lumbopelvines Alignment, Diversität (Abb. 4)

Die in Abb. 3a gezeigten Durchschnittswerte dürfen nicht darüber hinwegtäuschen, dass die Pelvic Incidence und damit die korrelierenden Werte des Sacral Slopes und des Pelvic Tilt erheblich streuen, wobei für die Pelvic Incidence Minimalwerte unter 20 ° und Maximalwerte über 80 ° beobachtet wurden.

**Figure 4:**
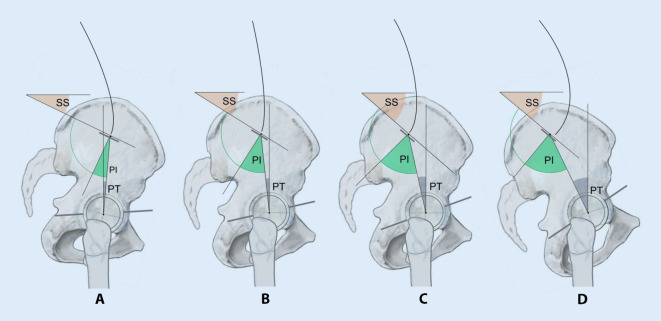

Es konnte mithilfe einer aufwendigen Studie [25] gezeigt werden, dass die Wirbelsäulenform eines Menschen von seiner individuellen Pelvic Incidence und deren korrelierendem Sacral Slope abhängt. Es sind hierbei – basierend auf dem Wert des jeweiligen Sacral Slopes – vier Formtypen zu unterscheiden, zu denen auch angegeben wurde, wie häufig der jeweilige Typ zu beobachten ist, und welche Typen bei welchen Wirbelsäulenerkrankungen zu beobachten sind. Analog zu diesen Roussouly-Formtypen der Wirbelsäule lassen sich anhand des Wertes der Pelvic Incidence und des Pelvic Tilt auch verschiedene Beckenversionen differenzieren, die sich klinisch unterschiedlich auswirken können:

- *Beckentyp A*: Dieser seltene Typ weist eine Pelvic Incidence deutlich unter 41 °und einen negativen Wert des Pelvic Tilt auf. Es besteht also eine extreme pelvine Retroversion, die sich bereits in der Beckenübersichtsaufnahme in Form einer „elephant’s ear hip“ [33] vermuten lässt. Der Hüftgelenksdrehpunkt ist dorsokaudal der Sakralbasismitte zu finden. Konsekutiv besteht fast immer eine azetabuläre Retroversion und damit ein femoroazetabuläres Impingement. Die Patienten sind zumeist einem Wirbelsäulentyp Roussouly 1 zuzuordnen, der durch einen Sacral Slope kleiner 35 °, eine kurze Lendenlordose und einen rückgeneigten Rumpf gekennzeichnet ist.
- *Beckentyp B*: Dieser „flat back-pelvic retroversion-type“ weist eine Pelvic Incidence unter 41 °und einen niedrigen Pelvic Tilt auf, der Hüftdrehpunkt liegt knapp ventral der Sakralbasis. Die Patienten weisen in der Regel einen Flachrücken auf, der dem Roussouly-Typ 2 zuzuordnen ist. Er findet sich überwiegend bei jungen Mädchen, die dann entweder über einen unspezifischen Kreuzschmerz [25] oder über beidseitige Leistenschmerzen klagen. Die Mehrzahl aller symptomatischen azetabulären Retroversionen sind diesem Beckentyp zuzuordnen [10].

Problem bei den Beckentypen A/B ist, dass bei diesen die Sakralbasis bereits sehr stark zur Horizontalen verlagert ist und die Lendenwirbelsäule sehr steil steht, sodass ein vorderes Hüftimpingement kaum über die Wirbelsäule kompensierbar ist.

- *Beckentyp C*: Dieser entspricht dem Normtyp der Abb. 4 mit einem Durchschnittswert der Pelvic Incidence von 52,0 ° ± 10,5 °, des Sacral Slopes von 40,2 ± 7,7° und des Pelvic Tilt von 11,6 ± 7,0°. Die Wirbelsäulenform ist dem Roussouly-Typ 3 zuzuordnen. Ein erhöhtes Risiko der Untersuchten, an einer sekundären Wirbelsäulen- oder Hüfterkrankung zu leiden, ist nicht gegeben.
- *Beckentyp D*: Bei diesem beträgt die Pelvic Incidence mehr als 62 °, das Hüftgelenk steht weit ventral der Sakralbasis und öffnet sich in der Regel stärker nach ventral.

Eine spezifische Hüfterkrankung für diesen Typ ist nicht bekannt, liegt jedoch gleichzeitig eine kongenitale Hüftdysplasie vor, verstärkt die pelvine Anteversion in ungünstiger Weise die Anteversion der Hüftpfanne [17]. Der korrelierend bestehende hohe Sacral Slope prädisponiert zur Spondylolisthesis [12].

## Funktionelle Änderungen der Beckenposition (Abb. 3b und 5)

Ein Individuum ist über genetische Mechanismen in seinem Alignmenttyp festgelegt, seine Beckenposition kann jedoch funktionell über die Vor- oder Rückrotation im Hüftgelenk (oder selten auch durch Beugestellungen im Kniegelenk) sekundär verändert werden. Der Wert der Pelvic Incidence bleibt hierbei immer gleich, die Werte des Sacral Slopes und des Pelvic Tilt ändern sich gegensinnig. Diese willkürliche oder durch Erkrankungen erzwungene Rotation im Hüftgelenksdrehpunkt, welche sekundär die Beckenposition verändert, wird in der Rehabilitationsmedizin, Sportmedizin und Physiotherapie als (funktioneller) anteriorer oder posteriorer Tilt bezeichnet [21].

**Figure 5:**
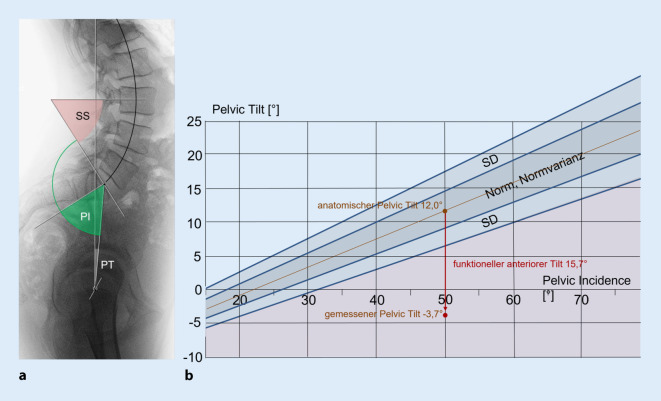

Gut verständlich sind die alltäglichen funktionellen Änderungen, wenn ein Individuum vom Stand zum Gehen, ins Sitzen und zum Liegen kommt. Beim Gehen ist in der Standbeinphase ein funktioneller anteriorer Tilt von 4 ° zu beobachten [22].

Beim Wechsel vom Stand zum Sitzen erhöht sich der Pelvic Tilt um circa 20–25°, der Sacral Slope erniedrigt sich um denselben Wert [13]. Beim Wechsel vom Stand zum Liegen vergrößert sich der Pelvic Tilt um durchschnittlich 2–6°, wobei große Spannbreiten zu beobachten sind [23]. Dies ist insbesondere in der Hüftarthroplastik und bei Beckenosteotomien zu beachten [7, 13, 14].

Bestimmt man den Pelvic Tilt eines Patienten, dann muss man sich dessen bewusst sein, dass sich der gemessene Wert aus dem anatomischen – mit der Pelvic Incidence korrelierenden – Tilt und einem überlagernden funktionellen Tilt zusammensetzt. Beide Komponenten sind mit einer „Eichkurve“ zu unterscheiden, die sich an großen Kollektiven gesunder Probanden orientiert [2, 16, 35]. Dies ist besonders eindrucksvoll bei neuroorthopädischen Erkrankungen zu sehen, die mit einer glutealen Schwäche oder Hüftbeugekontraktur einhergehen. In der Altersorthopädie sind es die degenerativ verursachte Lendenkyphose und die koxarthrotisch ausgelöste Hüftbeugekontraktur, die die Werte aus der anatomischen Normkurve ausscheren lassen.

## Lumbopelvines Alignment und azetabuläre Orientierung (Abb. 6)

Die Hüftpfanne entspricht geometrisch einem unregelmäßig berandeten Kugelsegment, dessen Eingangsebene und Polachse innerhalb des Beckens in erster Linie nach kaudolateral und weniger ausgeprägt nach ventrokaudal ausgerichtet ist [3, 20]. Diese komplexe Stellung im Raum kann man vereinfacht darstellen, indem man die Position der Pfanne mit dem Wertepaar der frontalen azetabulären Inklination und der sagittalen azetabulären Ante‑/Retroversion (Anteinklination) beschreibt [7, 13, 14] oder beide Komponenten miteinander verrechnet [20]. Die frontale Inklination der Pfanne lässt sich radioanatomisch problemlos mit dem Inklinationswinkel nach Ullmann vermessen [34]. Die sagittale Version der Pfanne wurde bisher – bei nicht validen Bezugspunkten in der seitlichen Beckenaufnahme – nur indirekt mithilfe von Surrogatparametern aus der Beckenübersicht [30], der axialen Computertomographie [1] oder der Faux-profile-Aufnahme [18] dargestellt. Inzwischen konnte die azetabuläre Anteversion mithilfe von 3D-Rekonstruktionen im EOS-System direkt bestimmt werden [32], wobei bei asymptomatischen Probanden Mittelwerte von 18 °gegenüber der Horizontalen angegeben wurden.

**Figure 6:**
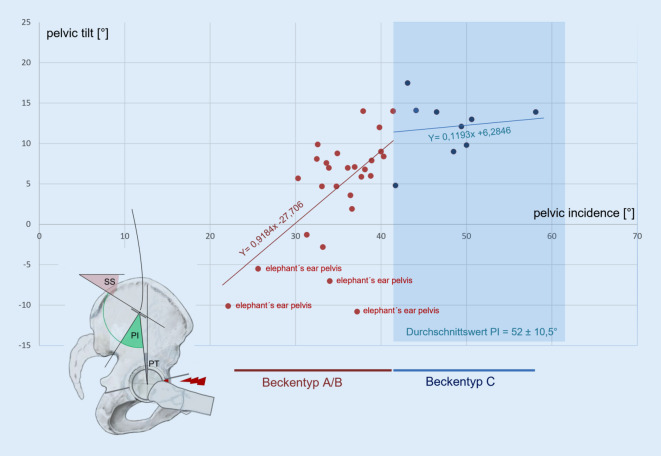

Im Rahmen der vorliegenden Übersicht interessiert, ob die sagittale azetabuläre Ante‑/Retroversion vom spinopelvinen Alignment beeinflusst ist, wie dies in Abb. 4 dargestellt ist, oder ob sich die Pfanne innerhalb des Beckens autonom orientiert. Hier gibt es Hinweise, dass Beckenformen mit niedriger Pelvic Incidence zur azetabulären Retroversion neigen, während solche mit hoher Pelvic Incidence stärker antevertierte Pfannen aufweisen und damit die azetabuläre Anteversion der kongenitalen Hüftdysplasie verstärken können [10, 17, 24, 32]. Eine lineare Korrelation zwischen der Pelvic Incidence und der azetabulären Orientierung wird jedoch bestritten [11]. In klinischen Arbeiten war zu beobachten, dass Patienten mit einer symptomatischen azetabulären Retroversion überwiegend niedrige Pelvic Incidences aufweisen [10, 17], dass es jedoch auch Patienten mit symptomatischer azetabulärer Retroversion und normaler Pelvic Incidence gibt [10].

## Fazit für die Praxis

- Die Wirbelsäulenform wie auch die pelvine Ante‑/Retroversion eines Individuums wird durch seine sagittale lumbosakrale Angulation bestimmt.
- Das lumbopelvine Alignment wird in der seitlichen Stehaufnahme des Patienten vermessen.
- Das sagittale Profil des Beckens kann funktionell über Vor- und Rückrotation im Hüftgelenk beeinflusst sein.
- Die Ausprägung des lumbopelvinen Alignments kann in vier verschiedenen Beckentypen A–D beschrieben werden.
- Der Typ A prädisponiert zum Krankheitsbild der azetabulären Retroversion und erfordert in der Hüftendprothetik eine modifizierte Pfannenpositionierung.
- Der Typ B beschreibt die Kombination eines ausgeprägten Flachrückens mit einer pelvinen Retroversion („flat back-pelvic retroversion-type“). Er prädisponiert zum nichtspezifischen Kreuzschmerz und/oder zum Krankheitsbild der azetabulären Retroversion.
- Der Typ D prädisponiert zur Spondylolisthesis und verstärkt die azetabuläre Anteversion von Patient:innen mit kongenitaler Hüftdysplasie.

## Abkürzungen

SS: „Sacral Slope“
PI: „Pelvic Incidence“
PT: „Pelvic Tilt“
